# Assessment of fluorescent protein candidates for multi-color flow cytometry analysis of *Saccharomyces cerevisiae*

**DOI:** 10.1016/j.btre.2022.e00735

**Published:** 2022-04-26

**Authors:** Raquel Perruca-Foncillas, Johan Davidsson, Magnus Carlquist, Marie F. Gorwa-Grauslund

**Affiliations:** Applied Microbiology, Department of Chemistry, Lund University, P.O. Box 124, SE-221 00 Lund, Sweden

**Keywords:** Fluorescent proteins, Multiple biosensors, Flow cytometry, Biosensors, Population heterogeneity, *Saccharomyces cerevisiae*

## Abstract

Transcription factor-based biosensors represent promising tools in the construction and evaluation of efficient cell factories for the sustainable production of fuels, chemicals and pharmaceuticals. They can notably be designed to follow the production of a target compound or to monitor key cellular properties, such as stress or starvation. In most cases, the biosensors are built with fluorescent protein (FP) genes as reporter genes because of the direct correlation between promoter activity and fluorescence level that can be measured using, for instance, flow cytometry or fluorometry. The expansion of available FPs offers the possibility of using several FPs - and biosensors – in parallel in one host, with simultaneous detection using multicolor flow cytometry. However, the technique is currently limited by the unavailability of combinations of FP whose genes can be successfully expressed in the host and whose fluorescence can be efficiently distinguished from each other.

In the present study, the broad collection of available FPs was explored and four different FPs were successfully expressed in the yeast *Saccharomyces cerevisiae*: yEGFP, mEGFP, CyOFP1opt and mBeRFPopt. After studying their fluorescence signals, population heterogeneity and possible interactions, we recommend two original combinations of FPs for bi-color flow cytometry: mEGFP together with either CyOFP1opt or mBeRFPopt, as well as the combination of all three FPs mEGFP, CyOFP1opt and mBeRFPopt for tri-color flow cytometry. These combinations will allow to perform different types of bi-color or possibly tri-color flow cytometry and FACS experiments with yeast, such as phenotype evaluation, screening or sorting, by single-laser excitation with a standard 488 nm blue laser.

## Highlights

• In vivo fluorescence of candidate fluorescent proteins was assessed in *Saccharomyces cerevisiae*.
• eEGFP, CyOFP1opt and mBeRFPopt were found to be suitable for multicolour flow cytometry.
• Successful protein selection and equipment configuration allowed potential tri-color flow cytometry with 488 nm single-laser excitation.

## 1 Introduction

Biobased production of fuels, chemicals and pharmaceuticals has become a key strategy in the development of more sustainable industrial processes. In this context, the yeast *Saccharomyces cerevisiae* has commonly been used as a platform organism due to its robustness to process conditions and the engineering opportunities offered through the numerous genetic tools available for this species. It is now possible to quickly generate thousands of enzyme and pathway combination variants to test for the production of a given compound in *S. cerevisiae*. However, when the best performing variants cannot be selected on the growth pattern, screening for the best performing strains remains a cumbersome task that slows down the overall process of obtaining optimal strains for microbial biobased production. One way to facilitate such screening is to use transcription factor-based biosensors [1] whose response is designed to be proportional to the production of the target compound. For example, Skjoedt et al., showed the possibility of real-time monitoring of *cis,cis*-muconic acid (CCM) production in yeast by introducing a transcription factor-based biosensor in which the transcriptional activator BenM controlled GFP expression [2]. Similarly, the production of acetic acid could be monitored by the development of a biosensor based on the transcription factor Haa1 [3]. Transcription factor-based biosensors have also been implemented in *S. cerevisiae* for measuring different cellular properties. One of the first examples was the DNA damage biosensor developed by Walmsley et al., in which GFP expression was controlled by the promoter of *RAD54*, a gene induced by exposure to UV, radiation or chemical agents such as methyl methanesulfonate [4]. Since then, a variety of biosensors have been developed to study cellular properties such as growth [5], redox state [6, 7], sugar sensing [8] or the unfolding response in protein production [9]. A recent compilation of developed biosensors in yeast can be found in [1].

In transcription factor-based biosensors, the expression of a reporter molecule is controlled by a promoter whose induction or repression depends on the binding of one or several transcription factors to the promoter region. Fluorescent protein (FP) genes are often used as reporter genes because the induced or repressed state of the promoter of interest can be directly correlated to fluorescence that is detectable using, for instance, flow cytometry or microscopy. Among them, the yeast enhanced green fluorescent protein (yEGFP) is the most commonly used reporter molecule in *S. cerevisiae*, due to its high fluorescence levels [10]. Following the discovery of the green fluorescent protein (GFP) [11], many other fluorescent proteins offering a wide range of colors have been identified and/or developed. These fluorescent proteins are often classified based on their emission wavelength range; thus, they are defined on the whole visible spectrum as (i) blue fluorescent proteins (BFP), (ii) cyan fluorescent proteins (CFP), (iii) green fluorescent proteins (GFP), (iv) yellow fluorescent proteins (YFP), (v) orange fluorescent proteins (OFP) and (vi) red fluorescent proteins (RFP) [12]. FPs have been used in a variety of applications from studies on host-parasite interactions – e.g., by obtaining GFP-expressing pathogenic bacteria and following their interactions with their hosts during infection- [13] to labeling of subcellular structures in mammalian cells [14]. Overall, a lot of FP studies still focus on biomedical applications with the use of in vivo imaging applying fluorescence microscopy.

The expansion of available FPs offers the possibility of using several FPs - and biosensors - in one host, with simultaneous detection using multicolor flow cytometry. If successful, the implementation of multicolor flow cytometry opens the possibility to investigate several cellular properties of interest at once as well as possible interactions between such properties. However, a major challenge of multicolor flow cytometry is the selection of the appropriate combination of FPs. Due to the limited space in the visible spectrum, the emission fluorescence of FPs overlap can make it difficult to distinguish between the emission signals [15]. Operational adjustments such as the selection of appropriate emission filters are also necessary to optimize the simultaneous detection of multiple fluorescence signals [16].

In the present study, the broad collection of available FPs was explored to identify and assess FPs of potential use as reporters in biosensor systems for the yeast *S. cerevisiae*. Several constructs carrying different FPs as reporter molecules under the expression of constitutive promoter TEF1p were generated and one copy of each of them was integrated into *S. cerevisiae* genome. The fluorescence activity of the obtained strains containing one of the selected FPs was analyzed using flow cytometry. Finally, possible novel combinations of FPs for multicolor flow cytometry were suggested and further evaluated.

## 2 Materials and methods

### 2.1 Strains and cultivation media

The *S. cerevisiae* strains and shuttle plasmids used and developed in the study are listed in Tables 1 and 2, respectively. For sub-cloning experiments, *Escherichia coli* NEB5α competent cells from New England Biolabs (Ipswich, MA, USA) were also used.

**Table 1:** Yeast strains used in the present study.

Name	Fluorescent protein	Relevant genotype	Reference
CEN.PK 113–7D	–	MATa URA3 HIS3 LEU2 TRP1 MAL2–8c SUC2	Euroscarf
Background strain	–	CEN.PK113–7D; pCfB2312	This study
TMBRP013	yEGFP	CEN.PK 113–7D; pCfB2312; XI-2::TEF1p-yEGFP-ADH1t	This study
TMBRP014	mEGFP	CEN.PK 113–7D; pCfB2312; XI-2::TEF1p-mEGFP-ADH1t	This study
TMBRP015	mHoneydew	CEN.PK 113–7D; pCfB2312; XI-2::TEF1p-mHoneydew-ADH1t	This study
TMBRP016	TagRFP657	CEN.PK 113–7D; pCfB2312; XI-2::TEF1p-yoTagRFP657-ADH1t	This study
TMBRP004	CyOFP1opt	CEN.PK 113–7D; pCfB2312; XI-3::TEF1p-CyOFP1opt-ADH1t	This study
TMBRP005	mBeRFPopt	CEN.PK 113–7D; pCfB2312; XI-3::TEF1p-mBeRFPopt-ADH1t	This study
TMBRP008	mEGFP+ CyOFP1opt	CEN.PK 113–7D; pCfB2312; XI-2::TEF1p-mEGFP-ADH1t; XI-3::TEF1p-CyOFP1opt-ADH1t	This study
TMBRP009	mEGFP+ mBeRFPopt	CEN.PK 113–7D; pCfB2312; XI-2::TEF1p-mEGFP-ADH1t; XI-3::TEF1p-mBeRFPopt-ADH1t	This study
TMBRP012	smURFPopt	CEN.PK 113–7D; pCfB2312; XI-3::TEF1p-smURFPopt-ADH1t	This study

**Table 2:** Plasmids used in the present study.

Name	Relevant genotype	Reference
YIpGFP	AmpR; URA3; yEGFP3-PGK1t	[8]
pNM001	pUC57; AmpR; TEF1p-ACS-PGK1t; GDPp-AT3-ADH1t	Unpublished
pCfB2312	pTEF1p-Cas9-CYC1t_kanMX	[24]
pCfB2903	XI-2 MarkerFree backbone	[24]
pCfB2904	XI-3 MarkerFree backbone	[24]
pCfB3044	gRNA_XI-2; natMX	[24]
pCfB3045	gRNA_XI-3; natMX	[24]
mEGFP-FKBP(M)x4	YIplac204; mEGFP	[18]
pNCS mHoneydew	mHoneydew	[19]
pFA6a-link-yoTagRFP657-Kan	yoTagRFP657	[20]
pRP001	pCfB2903; TEF1p-yEGFP3-ADH1t	This study
pRP002	pCfB2903; TEF1p-mEGFP-ADH1t	This study
pRP003	pCfB2903; TEF1p-mHoneydew-ADH1t	This study
pRP004	pCfB2903; TEF1p-yoTagRFP657-ADH1t	This study
pRP005	pCfB2904; TEF1p-yEGFP3-ADH1t	This study
pRP008	pCfB2904; TEF1p-CyOFP1opt-ADH1t	This study
pRP009	pCfB2904; TEF1p-mBeRFPopt-ADH1t	This study
pRP013	pCfB2904; TEF1p-smURFPopt-ADH1t	This study

Liquid cultures of *E. coli* were performed in Lysogeny Broth (LB) medium containing 10 g/l tryptone, 5 g/l yeast extract, 5 g/l NaCl, pH 7.0. Selection of successful transformants was done in LB agar plates (LB + 15 g/l agar) supplemented with ampicillin (50 mg/l) and incubated overnight at 37 °C.

Yeast strains were grown in Yeast Peptone Dextrose (YPD) medium containing 20 g/l peptone, 10 g/l yeast extract and 20 g/l glucose. Cultivations were performed at 30 °C and 180 rpm. Selection of transformants was done at 30 °C in YPD agar plates (YPD + 15 g/l agar) supplemented with geneticin (200 mg/l) and nourseothricin (100 mg/l) to select for the Cas9-kanMX and the gRNA-natMX plasmids, respectively.

**Table 3:** Compilation of fluorescent protein candidates and properties. Excitation peak (Exc peak) and emission peak (Em peak) are the wavelengths at which the excitation or emission is maximum, respectively. The brightness is a calculated value used for comparison which is the product of the extinction coefficient (strength of light absorbance) and quantum yield (efficiency of conversion of absorbed light into emitted light) of the fluorescent protein.

Name	Exc peak (nm)	Em peak (nm)	Brightness	Color classification	Organism origin	Oligomerization	Reference
yEGFP/mEGFP	488	507	33.6 (mEGFP)	Green (GFP)	*Aequorea victoria*	Polymer/Monomer	[17,18]
mHoneydew	487	562	2.04	Yellow (YFP)	*Discosoma* sp.	Monomer	[19]
CyOFP1	497	589	30.4	Orange (OFP)	*Entacmaea quadricolor*	Monomer	[21]
mBeRFP	446	611	17.55	Red (RFP)	*Entacmaea quadricolor*	Monomer	[22]
TagRFP657	611	657	3.4	Red (RFP)	*Entacmaea quadricolor*	Monomer	[20]
smURFP	642	670	32.4	Red (RFP)	*Trichodesmium erythraeum* IMS101	Monomer	[23]

### 2.2 Plasmid construction

For the plasmids carrying the tetrameric yeast-adapted GFP gene (yEGFP; [17]), the TEF1p-yEGFP3 fragment was obtained by overlap extension PCR with primers *TEF1_f* and *TEF1p-yEGFP_r_OE* (using pNM001 as template) and the primers *TEF1p-yEGFP_f_OE* and *yEGFP_r_SfaAI* (using YIplac211+yEGFP3 as template) (See Supplementary Table 1 for primer list). Plasmids pRP001 and pRP005 were then obtained by introducing the *TEF1p-yEGFP3* amplified fragment into pCfB2903 and pCfB2904, respectively, using PstI/SfaAI restriction sites. These plasmids were then used as a backbone in which the yEGFP encoding gene was exchanged by other candidates using XhoI/SfaAI restriction sites.

The gene encoding for the monomeric yeast-adapted GFP (mEGFP; [18]) was amplified from mEGFP-FKBP(M)x4 plasmid that was a gift from Benjamin Glick (Addgene plasmid # 85,004) using the *TEF1p-mEGFP_f_OE* and *mEGFP_r_SfaAI* primers (Suppl. Table 1). The obtained fragment was digested with XhoI and SfaAI and ligated into the linearized pRP001 using T4 DNA ligase (Thermo Fisher Scientific, Waltham, MA, USA).

The same approach was used for the genes encoding mHoneydew [19] and yoTagRFP657 [20]. In the case of mHoneydew, the gene was amplified from the plasmid pNCS mHoneydew, which was a gift from Erik Rodriguez & Roger Tsien (Addgene plasmid # 91,760) using the *YFP_f_XhoI* and *YFP_r_SfaAI* primers (Suppl. Table 1). yoTagRFP657 was amplified from the plasmid pFA6a-link-yoTagRFP657-Kan, which was a gift from Wendell Lim & Kurt Thorn (Addgene plasmid # 44,955) using the *RFP_f_XhoI* and *RFP_r_SfaAI* primers (Suppl. Table 1).

The genes encoding CyOFP1 [21], mBeRFP [22] and smURFP [23] were codon-optimized and purchased from GenScript (Piscataway, NJ, United States). Codon-optimization was designed using the GeneArt tool from ThermoFisher and XhoI and SfaAI restriction sites were added at 5′ and 3′ ends respectively. The genes were extracted from the GenScript plasmid by cleaving with XhoI and SfaAI and further ligated into linearized pRP005.

### 2.3 Yeast strain engineering

*S. cerevisiae* strains were generated using the CRISPR/Cas9 system developed by Jessop-Fabre et al., [24]. The cells were prepared and transformed using the high-efficiency LiAc protocol [25]. The background strain, a CEN.PK113–7D containing pCfB2312, was transformed with the plasmids pRP001, pRP002, pRP003, pRP004, pRP008, pRP009 and pRP013 linearized with NotI. This led to the generation of seven strains, each carrying one chromosomally integrated copy of the gene encoding for the respective FPs.

For the strains containing two different FPs, the gene encoding mEGFP was introduced in the already existing TMBRP004 (CyOFP1opt) and TMBRP005 (mBeRFPopt) by linearization of pRP002 with NotI.

The verification of transformants was done by colony PCR. Verification of proper integration of the FP in the XI-2 locus was performed using a primer annealing downstream of the integration site and an internal primer annealing on the FP. Primers *yEGFP_f_ver* and *XI-2_ver_r* were used to verify CEN.PK+pRFP1 transformants, primers *mEGFP_f_ver* and *XI-2_ver_r* were used to verify mEGFP integration in TMBRP014, TMBRP008 and TMBRP009, primers *YFP_f_ver* and *XI-2_ver_r* were used to verify mHoneydew integration in TMBRP015, primers *RFP_f_ver* and *XI-2_ver_r* for verification of yoTagRFP657 integration in TMBRP016 transformants and primers *smURFPopt_f_ver* and *XI-3_ver_r* for verification of smURFPopt integration in TMBRP012 transformants. In the case of integration of CyOFP1opt and mBeRFPopt, the verification was performed with two combined PCRs. First, the presence of the FP was verified using primers *CyOFP1_opt_f* and *CyOFP1_opt_r* for TMBRP004 and TMBRP008 whereas primers *mBeRFP_opt_f* and *mBeRFP_opt_r* were used for TMBRP005 and TMBRP009. Then, primers *XI-3_ver_f* and *XI-3_ver_r,* which anneal upstream and downstream of the integration site XI-3, were used to verify the location of the integration. Two positive transformants per generated strain were saved in glycerol stock and used for further experiments.

### 2.4 Flow cytometry experiments

All strains were grown in 250 ml baffled shake flasks containing 25 ml YPD and with a starting OD_620_ of 0.5. Samples were taken after 2, 3, 4, 5, 6, 7 and 24 h of cultivation. For in vivo stability studies, all strains were cultivated as mentioned above and the protein synthesis inhibitor, cycloheximide (10 µg/ml) or nourseothricin (200 µg/ml), was added after two hours of cultivation.

Flow cytometry measurements were performed using a BD Accuri C6 flow cytometer equipped with a BD Csampler (BD Biosciences, Franklin Lakes, NJ, USA). The detection filters 510/15 nm, 585/40 nm, 610/20 nm and 675/25 nm were used to collect fluorescence emissions. All detection filters were purchased from BD Biosciences (Franklin Lakes, NJ, USA).

Phosphate-buffered saline (PBS) was used to dilute the samples to OD_620_ < 1.0 when necessary. For each sample, 10,000 events were recorded at medium speed (35 μL/min). A threshold of 80,000 in forward scatter height (FSC—H) was applied to avoid background noise. A washing step was performed between each sample to avoid cross-contamination. The data analysis was performed using FlowJo™ v10.8.1 software (BD Life Sciences). Compensation was performed for FL1-H, FL2-H and FL3-H parameters using FlowJo's compensation tool. Samples used as references for the compensation were TMBRP014 (mEGFP) for FL1-H, TMBRP004 (CyOFP1opt) for FL2-H and TMBRP005 (mBeRFPopt) for FL3-H. The background strain was used as negative control for all three parameters. The compensation matrix obtained was applied to the samples when applicable.

## 3 Results

### 3.1 Selection of fluorescent protein candidates

A literature search was carried out to find fluorescent protein candidates that could be combined in *S. cerevisiae* with the well-known yEGFP to perform multiple fluorescence combinations. The criteria for the selection of the most suitable candidates were (i) the ability to be excited with the commonly found in flow cytometer lasers of 488 nm or 640 nm, (ii) the compatibility of the emission spectrum with GFP and preferably other candidates on the list and (iii) the suitability for recombinant expression in *S. cerevisiae* (Table 3).

**Fig. 1:**
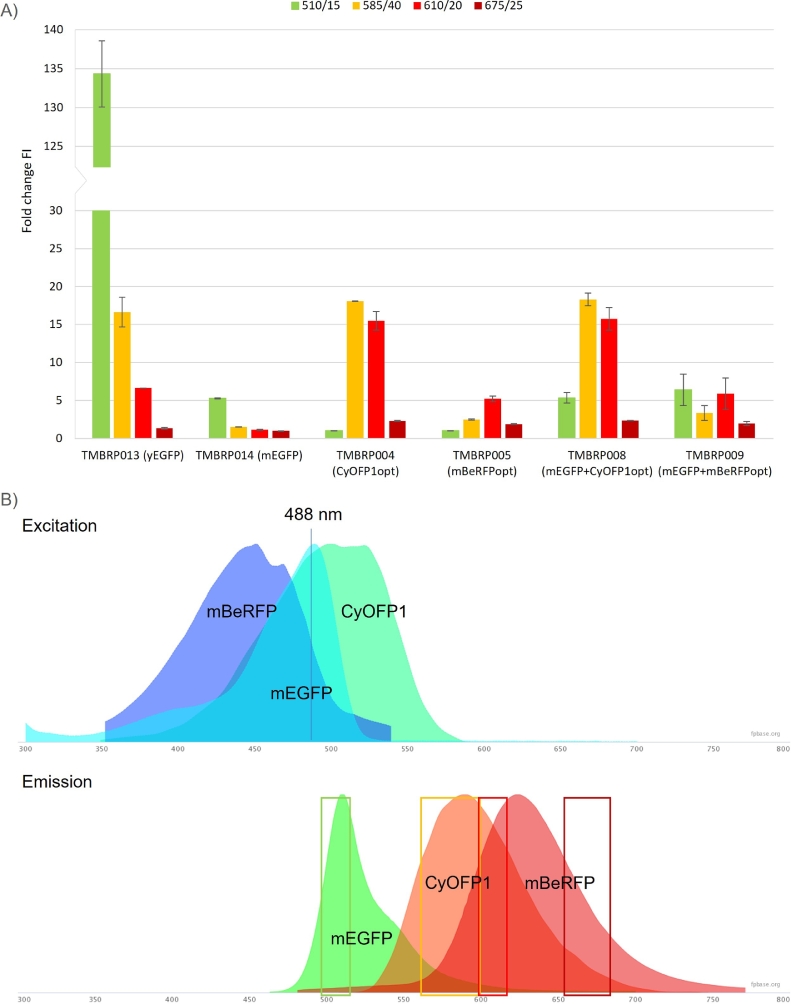
Fold change in emission fluorescence intensity (FI) for strains expressing different fluorescent proteins (FP), as compared to the background strain without any FP (A). Four FI were recorded for each strain corresponding to the four collection channels. From left to right: Green, fluorescence filter 510/15 nm; Yellow, fluorescence filter 585/40 nm; Light red, fluorescence filter 610/20 nm; Dark red, fluorescence filter 675/25 nm. Excitation and emission spectra of the fluorescent proteins mEGFP, CyOFP1opt and mBeRFP together with the experimental flow cytometry set-up: 488 nm excitation laser (blue line), 510/15 nm detection filter (green rectangle), 585/40 nm detection filter (yellow rectangle), 610/20 nm detection filter (red rectangle) and 675/25 nm detection filter (dark red rectangle) (B). Image adapted from the online spectra viewer tool in FPBase [26].

Six candidates were identified from FPbase [26] (Table 3). In addition to the well-known yeast-enhanced GFP, yEGFP, a monomeric variant of GFP, mEGFP, was selected. In the emission spectrum for YFPs, the protein mHoneydew was selected based on its potentially differentiable emission wavelength (562 nm) from EGFP (507 nm). CyOFP1 was selected among the OFPs because of its high brightness and its broad excitation spectrum (see Fig. 1B) that gives the possibility to be excited with a 488 nm laser with 96% of peak efficiency. Finally, three different RFPs were selected. The first one, mBeRFP, has its excitation peak at 446 nm making its excitation with a 488 nm blue laser non-optimal, but still achievable with a 47% excitation efficiency. The other two RFPs, TagRFP657 and smURFP, can be excited using a standard 640 nm red laser instead. Both of them have the emission peak in the far-red region of the spectrum. No BFP was selected, due to the absence of a suitable laser and filter configuration in the in-house flow cytometer.

### 3.2 Assessment of in vivo fluorescence in S. cerevisiae

In order to assess the fluorescence of the selected FPs in vivo in yeast, seven laboratory strains carrying each one FP were constructed: TMBRP013 (yEGFP), TMBRP014 (mEGFP), TMBRP015 (mHoneydew), TMBRP016 (yoTagRFP657), TMB RP004 (CyOFP1opt), TMB RP005 (mBeRFPopt) and TMB RP0012 (smURFPopt). Each strain contained one copy of the respective FP-encoding gene integrated into the genome under the expression of constitutive *TEF1* promoter, to ensure a comparison of fluorescence between FPs that are produced from similar expression levels. Two different loci were used for integration of the FPs, yEGFP, mEGFP, mHoneydew and yoTagRFP657 were integrated into XI-2 whereas CyOFP1opt, mBeRFPopt and smURFPopt were integrated into XI-3. To ensure that the integration site had limited impact on the observed fluorescence signal strength, yEGFP was integrated in parallel in both loci as a control. The results showed no significant difference in fluorescence since a 140.4-fold and 142.2-fold increase was detected for the integration in XI-2 and XI-3, respectively.

Fluorescence was measured after 3 h in yeast cells grown on YPD medium using the following non-standard detection filters configuration on the flow cytometer instrument: 510/15 nm on FL1 position, 585/40 nm on FL2 position, 610/20 nm on FL3 position and 675/25 nm on FL4 position. This configuration was designed to minimize spillover between recorded signals and it enabled the visualization of potential overlapping signals from the same FP on different detection filters. The strains TMBRP015, TMBRP016 and TMBRP003, carrying mHoneydew, yoTagRFP657 and smURFP respectively, showed very low or no fluorescence (data not shown) and were not further considered. The results for the other four strains, TMBRP013 (yEGFP), TMBRP014 (mEGFP), TMBRP004 (CyOFP1opt) and TMBRP005 (mBeRFPopt), are presented in Fig. 1A. The strains TMBRP013 carrying yEGFP and TMBRP014 carrying mEGFP showed high fluorescence emission at 510/15 nm (green channel) with a 134.3-fold and 5.3-fold increase in fluorescence intensity, respectively, as compared to the background strain without any FP. Due to the very high fluorescence level for yEGFP, non-negligible fluorescence also spilled over to the neighbouring 585/40 nm (yellow channel) and 610/20 nm (orange channel) detection filters. Fluorescence from the yeast codon-optimized version of CyOFP1 expressed in TMBRP004 could be detected both at 585/40 nm (yellow channel) and 610/20 nm (orange channel) with a wide dynamic range. An 18.1-fold and 15.5-fold increase in fluorescence were observed at 585/40 nm and 610/20 nm, respectively. In the case of the yeast codon-optimized version of mBeRFP (strain TMB RP005), a clear signal was observed at 610/20 nm (orange channel) with a 5.2-fold increase in fluorescent intensity, with limited spill-over in the neighbouring detection filters.

The selected equipment configuration allowed the measurement of four FPs, yEGFP, mEGFP, CyOFP1opt and mBeRFPopt. Despite not being optimal for all the FPs, the four FPs could be excited by the 488 nm blue laser (Fig. 1B), thus making only such excitation laser necessary. The excitation efficiency for mBeRFP at 488 nm was non-optimal with a 47% excitation efficiency and further improvements could be achieved if desired, i.e., with the addition of a second blue excitation laser at 458 nm which would increase its excitation. Nevertheless, the excitation obtained with the 488 nm laser was enough to obtain a distinct signal.

### 3.3 Combination of several fluorescent proteins in the same strain

For combinations of FPs to be suitable, the fluorescence intensity observed in all channels had to be considered to avoid significant overlapping of signals from different FPs in the same channel. The yEGFP signal was so strong that it gave fluorescence recorded at 585/40 nm (FL2, yellow channel) and at 610/20 nm (FL3, orange channel), thereby potentially masking the signals obtained from CyOFP1opt or mBeRFPopt that deliver a much weaker signal than the yEGFP one. Instead, the lower mEGFP only gave a significant signal at 510/15 nm leaving the other channels free to use for measuring the other FPs. Therefore, mEGFP was considered to be a better option for combinations as compared to yEGFP, even though the signal at 510/15 nm (FL1, green channel) was much stronger for yEGFP.

CyOFP1opt could be detected both at 585/40 nm and at 610/20 nm, but there was no signal at 510/15 nm (Fig. 1A). Thus, a combination of mEGFP and CyOFP1opt was of interest. Similarly, mBeRFPopt showed high fluorescence increase at 610/20 nm but no signal at 510/15 nm enabling the combination of mBeRFPopt with mEGFP. Consequently, two new strains were constructed in which these two combinations of FPs were attempted: TMBRP008 (mEGFP+CyOFP1opt) and TMBRP009 (mEGFP+mBeRFPopt). The signals, obtained under the same conditions as above, for TMBRP008 (mEGFP+CyOFP1opt) were consistent with those of TMBRP014 (mEGFP) and TMBRP004 (CyOFP1opt) separately (see Fig. 1A). For mEGFP, a 5.3-fold increase was detected in the single FP strain CEN.PK+pRPF2 (mEGFP) at 510/15 nm (Fig. 1A) compared to a 5.8-fold increase in the case of the double FP TMBRP008 (mEGFP+CyOFP1opt) (Fig. 1A). For the orange FP (CyOFP1opt), a similar 18.1- and 18.9-fold increase in fluorescence was detected at 585/40 nm, for TMBRP004 (CyOFP1opt) and TMBRP008 (mEGFP+CyOFP1opt), respectively. With the other detection filter (610/20 nm), close values of 15.5-fold versus 16.8-fold respectively were also recorded for the same two strains, whereas no significant fluorescence signal was detected at 675/25 nm (red channel). Due to the broad excitation of CyOFP1opt, the emission from mEGFP could be a source of excitation for CyOFP1opt when expressed together (see Fig. 1B), however, this was not observed confirming that no interference was produced between both fluorescence proteins.

A similar pattern was observed when assessing the combination of mEGFP with the red FP mBeRFPopt. In this case, the values observed were slightly higher with a 7.9-fold increase at 510/15 nm (green channel) for TMBRP009 (mEGFP+mBeRFPopt), compared to the 5.3-fold increase showed by TMBRP014 (mEGFP). At 610/20 nm (orange channel), TMBRP005 (mBeRFPopt) showed a 5.2-fold induction whereas TMBRP009 (mEGFP+mBeRFPopt) reported a 7.4-fold increase in fluorescence.

### 3.4 Differentiation of FP signals

**Fig. 2:**
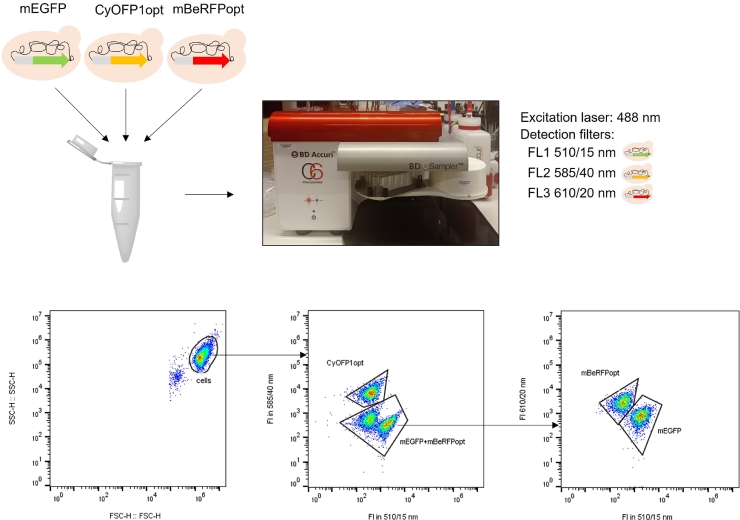
Experimental set-up and analysis for the design of a gating strategy by a mixed sample containing strains TMBRP014 (mEGFP) (green), TMBRP004 (CyOFP1opt) (orange) and TMBRP005 (mBeRFPopt) (red).

To further investigate the interaction between the FPs mEGFP, CyOFP1opt and mBeRFPopt, a study of the distribution of the populations was performed. A sample containing a mixture of three strains, TMBRP014 (mEGFP), TMBRP004 (CyOFP1opt) and TMBRP005 (mBeRFPopt), was analyzed (Fig. 2). First, the strains were analyzed separately and their concentration was quantified. This allowed generating a mixture of the three strains with the same number of cells for each strain. An initial selection of cells in the sample was made based on size by plotting forward scatter height (FSC—H) vs side scatter height (SSC—H). The fluorescence of the cells was analyzed by plotting FI at 510/15 nm vs FI at 585/40 nm which allowed the clear identification of the CyOFP1opt-carrying population due to its high FI at 585/40 nm. Then the rest of the cells, designated as mEGFP+mBeRFP, were plotted FI at 510/15 nm vs FI at 610/20 nm to make the distinction between both populations clearer. The mBeRFPopt-carrying population was identified by its high FI at 610/20 nm whereas a high signal at 510/15 nm was detected for the mEGFP-carrying population. The population distribution obtained by the gating strategy was 31.8% for TMBRP014 (mEGFP), 33.2% for TMBRP004 (CyOFP1opt) and 30.4% for TMBRP005 (mBeRFPopt), which was in agreement with the expected 33% for each of the strains.

**Fig. 3:**
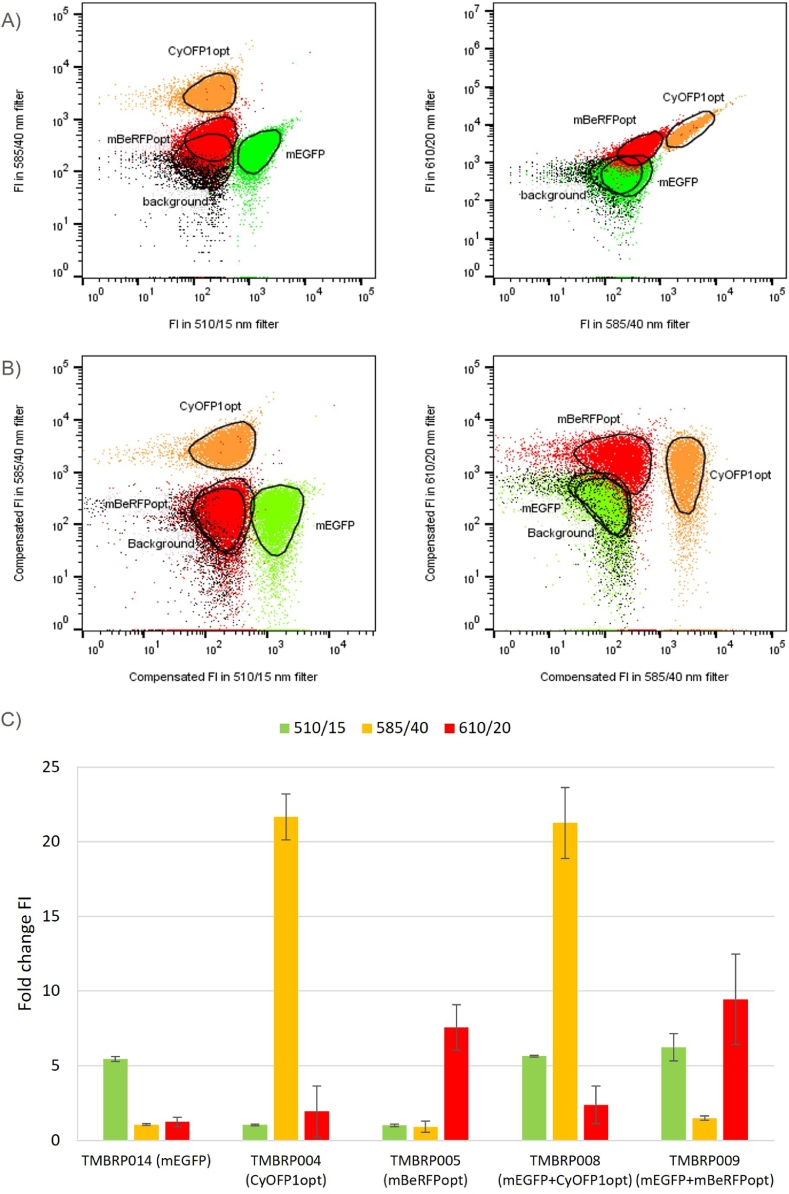
Dot plot showing the location of the background strain (black), TMBRP014 (mEGFP) (green), TMBRP004 (CyOFP1opt) (orange) and TMBRP005 (mBeRFPopt) (red) before compensation (A) and after compensation (B) in filters 510/15 nm vs 585/40 nm (left) and filters 585/40 nm vs 610/20 nm (right). Fold change in emission fluorescence intensity (FI) for strains expressing different fluorescent proteins (FP), as compared to the background strain without any FP after compensation (C). Four FI were recorded for each strain corresponding to the four collection channels. From left to right: Green, fluorescence filter 510/15 nm; Yellow, fluorescence filter 585/40 nm; Light red, fluorescence filter 610/20 nm; Dark red, fluorescence filter 675/25 nm.

Although the strains' fluorescence emissions were distinguishable from each other and a gating strategy for identification of each of the FPs signal was designed (Fig. 2), further improvements were attempted. Distributions shaped diagonally were observed, especially in CyOFP1opt for detection filters 585/40 nm vs 610/20 nm (Fig. 3A); this happens when spillover occurs i.e., CyOFP1opt signal being recorded in the red channel (610/20 nm filter). To avoid this problem, compensation of FL1-H, FL2-H and FL3-H parameters was performed using FlowJo's compensation tool. Samples containing individual FPs were used as positive controls: mEGFP for FL1-H (510/15 nm), CyOFP1opt for FL2-H (585/40 nm) and mBeRFP for FL3-H (610/20 nm). In contrast, the background strain was used as a negative control for all parameters. As a result, the fluorescences were corrected and the diagonal appearances of the FI distributions were no longer visible (Fig. 3B). The outcome of the compensation was further tested by recalculating the fold-change in FI observed in the different strains with the compensated values for FI after applying the compensation matrix (Suppl. Table 2). This led to only one signal that was clearly visible in the strains expressing one FP TMBRP014 (mEGFP), TMBRP004 (CyOFP1opt) and TMBRP005 (mBeRFPopt) in their corresponding detection filters, 510/15 nm, 585/40 nm and 610/20 nm, respectively (Fig. 3C). Likewise, TMBRP008 (mEGFP+CyOFP1opt) showed two clear signals at 510/15 nm and 585/40 nm whereas TMBRP009 (mEGFP+mBeRFPopt) showed them at 510/15 nm and 610/20 nm. As a consequence of compensation, a reduction in fold-change FI in the red channel was observed for the strains carrying CyOFP1opt, TMBRP004 and TMBRP008 as opposed to the values before compensation (Fig. 1A). These results confirmed the compatibility between the fluorescent proteins making the combination of mEGFP, CyOFP1opt and mBeRFPopt a good candidate for simultaneous measurement of three fluorescence emissions in flow cytometry.

### 3.5 Fluorescent protein impact and properties

**Fig. 4:**
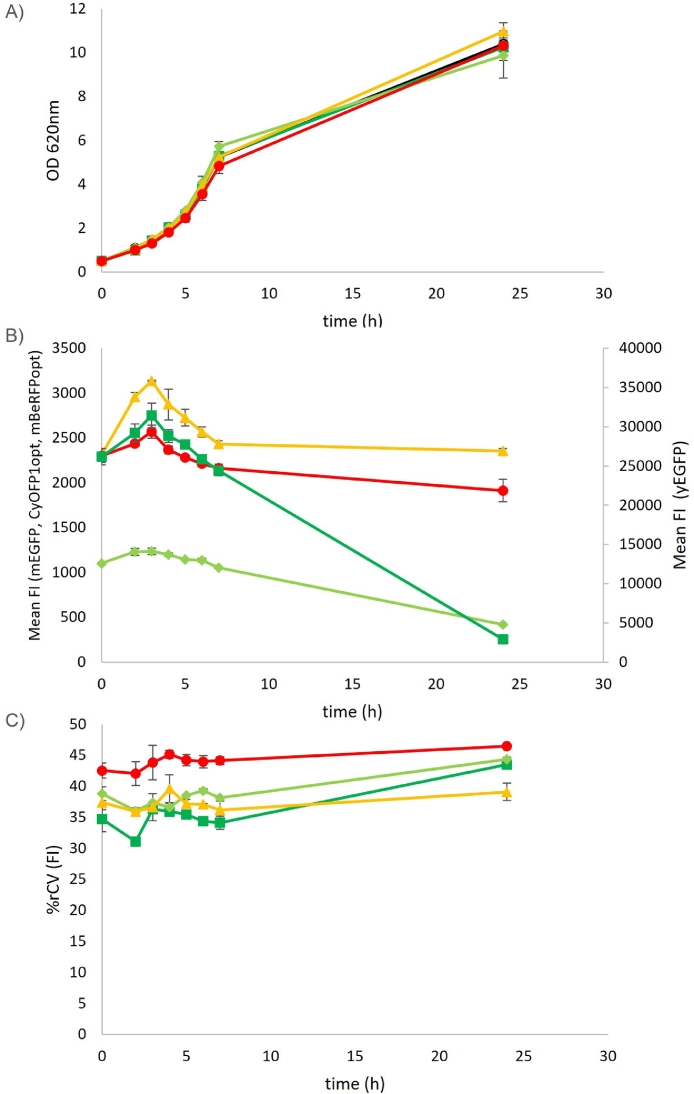
Optical density (A), mean fluorescence intensity (B) and robust coefficient of variation (%) of fluorescence intensity (C) over time for the background strain (black,○) and the constructed strains TMBRP013 (yEGFP) (dark green, ■), TMBRP014 (mEGFP) (light green, ♦), TMBRP004 (CyOFP1opt) (yellow, ▲) and TMBRP005 (mBeRFP) (red, ●). Fluorescence emissions for TMBRP013 (yEGFP) (dark green, ■) and TMBRP014 (mEGFP) (light green, ♦) were detected in the 510/15 nm filter corresponding to the green channel; TMBRP004 (CyOFP1opt) (yellow, ▲) in the 585/40 nm filter corresponding to the yellow channel; and TMBRP005 (mBeRFPopt) (red, ●) in the 610/20 nm filter corresponding to the orange channel. The fluorescence intensity of the background strain was below 500 for all three filters in all measured time points and was excluded from the figure for simplicity.

Possible interference caused by the expression of the introduced FPs was assessed by growth experiments on YPD medium. The addition of the fluorescent reporters showed no effect on the growth pattern for all the constructed strains, TMBRP013 (yEGFP), TMBRP014 (mEGFP), TMBRP004 (CyOFP1opt) and TMBRP005 (mBeRFPopt), as compared to the background strain (Fig. 4A). This highlighted the non-invasiveness of the introduced reporter(s). Morphological changes in the cell population were studied by looking at the forward scatter (FSC—H), which correlates with the size of the cells. A pattern corresponding to the budding processes was observed where the FSC—H initially increased in exponential phase and decreased towards the stationary phase (Suppl. Fig. 1). This pattern was observed for all strains, i.e. independently from the used FPs, indicating that the expression of FPs did not infer any morphological changes.

Next, the response of the four tested FPs, yEGFP, mEGFP, CyOFP1opt and mBeRFPopt expressed under the constitutive *TEF1* promoter, was followed over time to map the dynamics of each fluorescence signal. From the study of Peng et al., in which *TEF1* promoter activity was followed over time by GFP expression in similar conditions (20 g/l of glucose) [9], an initial increase in fluorescence was expected during the fermentative phase, followed by a decrease initiated during the diauxic shift which continued during the ethanol consumption phase, reaching low levels after 24 h. In the present experiment, a similar pattern was observed for all four FPs during the first seven hours, with an increase in fluorescence that reached its highest point at 3 h of cultivation (Fig. 4B); from this point, the fluorescence decreased progressively. However, a main difference could be observed after 24 h. In strains carrying GFPs, mEGFP and yEGFP, an abrupt decrease of the fluorescence was observed whereas the fluorescence in strains carrying CyOFP1opt and mBeRFPopt stabilized and remained high after 24 h of cultivation.

**Fig. 5:**
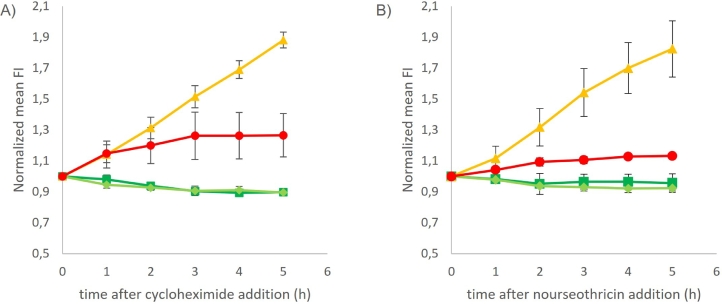
Normalized mean fluorescence intensity after addition of protein synthesis inhibitor cycloheximide (A) or nourseothricin (B) for TMBRP013 (yEGFP) (dark green, ■) and TMBRP014 (mEGFP) (light green, ♦) in the 510/15 nm filter corresponding to the green channel; TMBRP004 (CyOFP1opt) (yellow, ▲) in the 585/40 nm filter corresponding to the yellow channel; and TMBRP005 (mBeRFPopt) (red, ●) in the 610/20 nm filter corresponding to the orange channel. Values were normalized to the initial fluorescent intensity to facilitate comparison.

To further assess the in vivo stability and half-life of the different FPs, a protein synthesis inhibitor was added to the medium and the evolution of the fluorescence signal was recorded from that point. Since new proteins could not be synthesized and no growth was possible either, it was assumed that the decrease in fluorescence would correspond to the degradation of the available protein. Cycloheximide was first used as a protein synthesis inhibitor as it is commonly used for half-life determination [27]. Both GFPs, mEGFP and yEGFP, showed a decrease in fluorescence after the addition of cycloheximide (Fig. 5A). Unexpectedly, CyOFP1opt and mBeRFPopt showed an increase in fluorescence intensity after cycloheximide addition (Fig. 5A). To elucidate whether the response was substance dependent or not, nourseothricin was tested as an alternative protein synthesis inhibitor. Nevertheless, the response was consistent with that of cycloheximide: both GFPs decreased in fluorescence and CyOFP1opt and mBeRFPopt fluorescence signals increased over time (Fig. 5B). Both GFPs, yEGFP and mEGFP, showed similar degradation patterns that were further confirmed with the estimation of a half-life of ca. 22 h in both cases by obtaining the slope of the linear regression fitting the linearized fluorescence curve. No calculation of half-life was possible for CyOFP1opt and mBeRFPopt, due to the unexpected behavior for these proteins that remains to be solved.

### 3.6 Fluorescent proteins for population heterogeneity studies

One of the strongest advantages of using flow cytometry, as compared to fluorometry, is the possibility of obtaining single-cell measurements that can give information on the heterogeneity of the studied population. In the present study, the distribution of fluorescence was gaussian like with a slight skewness to higher fluorescence, and no subpopulations with low fluorescence level arising from dead cells or poor expression was observed.

Distribution of FP expression was assessed by the coefficient of variance (CV), which is the ratio between the standard deviation of the distribution and its mean fluorescence. Similarly, the robust coefficient of variance (rCV), that is less skewed by possible outliers in the population, was used. All strains expressing the different FPs showed similar rCV values, ranging from 31% to 46% rCV, which suggest that the expression of the fluorescent proteins yEGFP, mEGFP, CyOFP1opt and mBeRFPopt were equally distributed within the population. The fluorescent protein mBeRFPopt showed a slightly higher rCV (p-value < 0.05) than the rest of the FPs (Fig. 4C). Furthermore, an increase in distribution level was observed at the end of the cultivation (24 h), especially in the case of yEGFP and mEGFP (Fig. 4C).

## 4 Discussion

The present study identifies original combinations of fluorescence proteins that can be used in the yeast *S. cerevisiae* for the simultaneous assessment of different cellular properties or to simultaneously screen for several phenotypes, for instance the production of different compounds that are recognized by corresponding biosensors.

Many FPs are available and databases such as FPbase offer a vast compilation of data from their FP collection including properties like emission spectrum, brightness or molecular weight [26] which facilitates the selection process of a FP of interest. However, most of the available information comes from in vitro studies from native proteins or proteins produced with a limited host range. This makes in vivo characterization of the FP in the intended microorganism a necessary step to study the suitability of said FP [28]. In our study, we demonstrate the need for this step, as seven types of FPs were selected for multicolour detection based on their emission spectra, but only four of them resulted in detectable fluorescence intensity when one copy of the corresponding gene was integrated and expressed in *S. cerevisiae*. The absence of a distinct signal in strains carrying mHoneydew (YFP) and the RFPs yoTagRFP657 and smURFP could be due to different factors. In the case of mHoneydew and yoTagRFP657, their previous expression in *S. cerevisiae* in a multicopy plasmid had been successful yielding detectable fluorescence intensities (Hagman, personal communication). However, the integration of one copy was not sufficient here to give a distinct fluorescence signal. This could arise from the low brightness, 2.04 and 6.4 for mHoneydew and yoTagRFP657, respectively, compared to the 33.6 of GFP. In the case of the RFP smURFP, on the other hand, the comparable brightness (32.4) to that of GFP makes it unlikely to be the cause of the absence of fluorescence. smURFP is evolved from a cyanobacterial phycobiliprotein that uses biliverdin as cofactor. Since smURFP has been successfully expressed in *E. coli* and mammalian cells [29], the challenges observed in *S. cerevisiae* may come from the absence of this cofactor. However, our first trials to add a multicopy plasmid carrying the corresponding HO-1 gene [29] to increase the biliverdin supply did not help in increasing the fluorescence signal in *S. cerevisiae* (data not shown).

Using flow cytometry and several FPs, we demonstrate that different populations can be detected within the same sample based on their fluorescence type. This is of interest in fluorescence microscopy, where tagging of multiple cellular components or fusion proteins using FPs can be followed; in that case, properties such as small size or high stability are desired for the chosen FPs to obtain translational fusions with minimal imprint and prolong their study over time due to their long half-lives. Instead, when developing biosensors for population screening or to follow the dynamics of phenotypic properties, the used FPs should have sufficient fluorescence intensity to offer a wide range of detection but shorter half-live times that could be used to report dynamic changes. Ideally, a dynamic biosensor should be able to report both induction where an increase of fluorescence would be observed and repression where a decrease in fluorescence would be expected. From our results, we can observe that the four FPs tested are too stable for dynamic reporting of down-regulation events. In the case of both GFPs, an estimated half-life of ca. 22 h was obtained. This result that differs from the previously reported half-life of ca. 7 h for GFP [30,31] could be resulting from the calculation method used or experimental conditions. In the case of CyOFP1opt and mBeRFPopt an estimation of their half-lives was unfortunately not possible to obtain due to the unexpected and unexplainable increase in fluorescence when exposed to protein synthesis inhibitors (Fig. 5). Initial hypotheses for the observed increase in FI were (i) the monomeric nature of these proteins, (ii) possible interactions with the inhibitor or (iii) the origin of the fluorescence in those detection filters (585/40 nm and 610/20 nm) being from cellular components and not the fluorescent protein. However, these were later discarded as (i) it was not the case for mEGFP, also a monomeric variant, (ii) two different inhibitors were tested and showed a similar pattern and (iii) no increase in those detection filters was observed for the strains carrying yEGFP or mEGFP. Further work to elucidate this behavior is, however, beyond the scope of the present study. Nevertheless, the fluorescence intensity remained constant after 24 h of cultivation (Fig. 4B), thus suggesting a much longer half-life than that of GFP. The long stability of FPs is a known issue that has been addressed by generating destabilized variants like yEGFP3-Cln2_PEST_ [31]. Although the half-life of yEGFP3-Cln2_PEST_ has been greatly decreased to ca. 30 min, it relies on the ubiquitin-dependent degradation system in yeast, which could potentially interfere with the cell metabolism rendering it unsuitable for non-invasive monitoring biosensor applications.

In our study, four different FPs were successfully expressed in *S. cerevisiae* and their fluorescence signals were detected with the objective of finding suitable combinations for simultaneous detection. The first one was the FP yEGFP that is widely used in yeast research, and specifically in transcription factor-based biosensors [10] since it offers a broad dynamic range due to its high levels of fluorescence. However, we show that yEGFP is not optimal for multicolour flow cytometry purposes due to its high spillover signal into the rest of the detection filters. Instead, its monomeric variant, mEGFP, still shows a strong green fluorescence signal without spilling over other detection filters, making it a better candidate for multicolour flow cytometry. The other two FPs, CyOFP1opt and mBeRFPopt, also gave reasonably high signal in their respective emission channels. Since a key aspect of multicolour flow cytometry is to minimize the overlapping of the different fluorochromes [32], we recommend two original combinations of FPs for bi-color flow cytometry: mEGFP together with either CyOFP1opt or mBeRFPopt. It is also possible to use the combination of all three FPs mEGFP, CyOFP1opt and mBeRFPopt for tri-color flow cytometry; in the latter case, the additional use of compensation methods is recommended to obtain much cleaner signals and avoid spillover.

Combinations of up to four FPs have already been achieved in *S. cerevisiae*, including the use of FPs such as mTagBFP, mCherry, TagRFP-T, CFP or YFP; however, these were all optimized for live-cell imaging studies [20,33,34]. While compiling the present study, a parallel study was also published with a combination of up to three FPs, mTurquoise2, mCherry and YmPET whose genes were co-expressed in *S. cerevisiae* and the fluorescence recorded using a biolector [15]. All these approaches have in common the need for several excitation lasers, including a violet (405 nm) or yellow (561 nm) laser which are not commonly available in commercial flow cytometers. In contrast, our approach uses the fluorescent proteins mEGFP, CyOFP1opt and mBeRFPopt, which are all excited with a 488 nm blue laser. This opens up the possibility of performing multicolour flow cytometry by single-laser excitation with a 488 nm excitation laser that is provided in all simple flow cytometers as the primary excitation source [35]. To develop combinations of multiple FPs in flow cytometry to its full potential, the secondary excitation source should also be used. Since a red laser is commonly used as secondary excitation source [35], the far-red region of the visible spectrum should be further exploited.

## Declarations of Competing Interest

None.

## Appendix Supplementary materials

Image, application 1
