# Supplementary material for: Assessment of fluorescent protein candidates for multi-color flow cytometry analysis of Saccharomyces cerevisiae
Source: Biotechnol Rep (Amst). 2022 Apr 26;34:e00735. doi: 10.1016/j.btre.2022.e00735 (PMC9171426; doi:10.1016/j.btre.2022.e00735)
Supplement: Supplementary file 1 [file mmc1.docx]

# Supplementary materials

**Supplementary Table 1**. List of primers used in the project. Bold letters indicate the introduction of a restriction site. Lower case letters indicate the segment annealing to a gene whereas upper case letters correspond to primer tails.

| Primer name | Sequence (5’ 🡪 3’) |
| --- | --- |
| TEF1p-yEGFP_r_OE | ACCTTTAGACATATA**CTCGAG**tttgtaattaaaacttagattagattgc |
| TEF1p-yEGFP_f_OE | TTTAATTACAAA**CTCGAG**TATatgtctaaaggtgaagaattattcac |
| yEGFP_r_SfaAI | TGAATA**GCGATCGC**ttatttgtacaattcatccataccat |
| TEF1p-mEGFP_f_OE | AATTACAAA**CTCGAG**TATatggtgagcaagggc |
| mEGFP_r_SfaAI | TGAATA**GCGATCGC**ttacttgtacagctcgtccat |
| YFP_f_XhoI | TACAAA**CTCGAG**Tatatggtgagcaagggcga |
| YFP_r_SfaAI | ATGCGT**GCGATCGc**ttacttgtacagctcgtcc |
| RFP_f_XhoI | TACAAA**CTCGAG**TATatgagcgaattgatcacaga |
| RFP_r_SfaAI | ATGCGT**GCGATCGc**ttaattgagtttatgacctagttt |
| yEGFP_f_ver | acataaccttctggcatggc |
| mEGFP_f_ver | gaacttcagggtcagcttgc |
| YFP_f_ver | cttggccatgtaggtggtct |
| RFP_f_ver | tcccacgtaaaaccttctgg |
| XI-3_ver_f | ggccgttatttgtgcttgat |
| XI-3_ver_r | cggttgtgatattgttcctgc |
| smURFPopt_f_ver | ggaactggaacacccaaaga |
| CyOFP1_opt_f | atggtgtctaagggtgaagag |
| CyOFP1_opt_r | tcacttgtacaactcatccatacc |
| mBeRFP_opt_f | atggtttctaaaggtgaagaat |
| mBeRFP_opt_r | ttatttatgacccaatttagatggc |
| TEF1_f | TCGTTCTTCCACACCTGCAGatagcttcaaaatgtttctactcc |
| XI-2_ver_r | actgggaacagaaatcgacc |

**Supplementary Table 2**. Compensation matrix obtained using FlowJo.

| Fluorescence protein | FL1-H  510/15 nm | FL2-H  585/40 nm | FL3-H  610/20 nm |
| --- | --- | --- | --- |
| mEGFP | 100 | 8,0293 | 7,4762 |
| CyOFP1opt | -0,3418 | 100 | 233,6872 |
| mBeRFPopt | 0,4243 | 12,7234 | 100 |


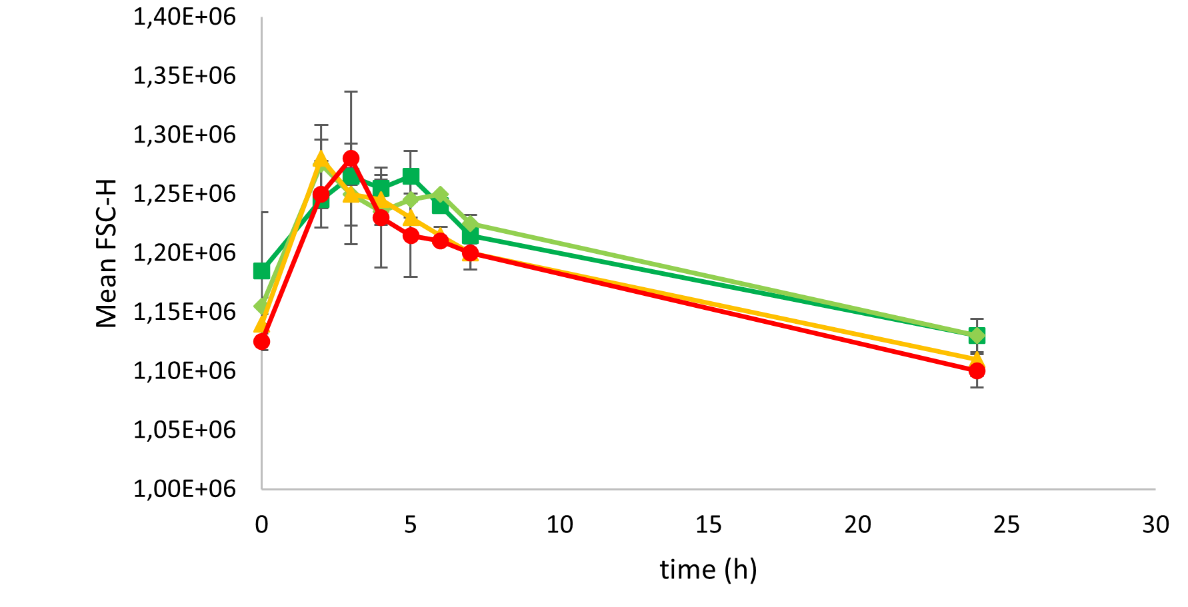


**Supplementary Figure 1**. Mean forward scatter height (FSC-H) over time for the constructed strains TMBRP013 (yEGFP) (■), TMBRP014 (mEGFP) (♦), TMBRP004 (CyOFP1opt) (▲) and TMBRP005 (mBeRFP) (●). Error bars represent standard deviations of biological duplicates.
